# Identifying Genes Involved in Innate Immunity through RNAi

**DOI:** 10.1371/journal.pbio.0020249

**Published:** 2004-06-22

An organism's ability to sense and respond to potentially harmful pathogens is key to its survival. To fight off disease and infection, organisms must detect pathogens, activate immune cell signaling pathways, and produce molecules able to thwart a pathogenic attack. So fundamental is this need that molecules and protein domains related to innate immunity are evident in organisms as diverse as plants, flies, and humans, highlighting the ancient origins of defense mechanisms. Once detected, the pathogen's presence triggers a cascade of signaling events that generate a rapid response tailored to specific classes of pathogens.

When pathogens attack the *Drosophila* fruitfly, they elicit a range of defensive reactions in the fly, including the production of antimicrobial proteins, phagocytes (which engulf the pathogen), and toxic metabolites. A microbe sets off one of these responses by interacting with a receptor, triggering a pathway that activates a special class of transcription factors, which in turn activate genes needed to make antimicrobial peptides, say, or toxins. *Drosophila* attacked by fungi and a certain class of bacteria activate these transcription factors through a pathway (the Toll pathway) that also operates in mammals.

A second pathway is activated when a different class of bacteria attack. While the general steps of this pathway, called the Immune deficiency (Imd) pathway, are known—Dredd-mediated activation of the Relish transcription factor, for example, is central to this antibacterial response—the details and mechanisms remain unclear. Signaling pathways are notoriously complex and the Imd pathway is no different. In the current model, bacterial pathogens stimulate a transmembrane receptor, which activates the Imd protein, which then transmits the signal through intermediary proteins, which ultimately activate Dredd, sending Relish into the nucleus to activate genes required for an immune response.

In this issue of *PLoS Biology*, Edan Foley and Patrick O'Farrell use a genome-wide approach to characterize the Imd pathway, with an eye toward understanding what regulates the Dredd-Relish interaction. To identify pertinent genes and their roles, Foley and O'Farrell took advantage of a technique, called RNA interference (RNAi), that can selectively target and “silence,” or inhibit, nearly any gene. After silencing a gene, researchers can then track how a cell responds and infer the gene's function. The technique uses double-stranded RNA (dsRNA) molecules with nucleic acid sequences that match a gene of interest (RNA can bind to DNA through complementary base pairing). The dsRNAs precipitate a series of steps that ultimately degrade the messenger RNA associated with the gene, preventing messenger RNA translation, thereby silencing the gene.

The authors produced over 7,000 dsRNAs corresponding to most of the *Drosophila* genes with counterparts in mammals or the worm *C. elegans* (that is, conserved genes), then developed a cell culture model to analyze the components of the Imd pathway. Since the cells in these cultures respond to the presence of bacterial proteins by generating antimicrobial peptides, they provide a good testbed for identifying genes involved in the pathway. Foley and O'Farrell's RNAi screen identified many molecules involved in signaling, including both signal inhibitors and activators. Among these signaling components, they discovered two new genes: one, which they named *sickie*, is required to activate Relish; the second, called *defense repressor 1 (dnr1)*, appears to inhibit Dredd activity and thus inhibit the pathway. Based on molecular analysis of these genes—which involved exposing cells to bacterial proteins—the authors propose a model of Imd signaling in which Dredd and the protein produced by *dnr1*, called Dnr1, operate through a negative feedback loop: Dredd activity appears to promote the accumulation of its own inhibitor, Dnr1. Since suppression of Dnr1 through RNAi can trigger an immune response, the authors explain, it appears that interruption of this feedback loop activates the signaling pathway. Without the inhibitory action of Dnr1, Dredd can activate Relish, which dissociates from Dredd, enters the nucleus, and activates the transcription of antimicrobial genes.

It's likely that the two genes described here play a key role in activating Relish, the authors conclude, and that others identified in this screen will also prove significant. Since many of these components are conserved in mammals, the pathway may likewise operate in humans. Future experiments will be the test of these assumptions, but until then, Foley and O'Farrell have demonstrated the soundness of using RNAi screens on a large scale to dissect the elements of a complex signaling pathway.

